# Exploratory Analysis of Serum IGF-I Levels and Symptom Trajectories in Long COVID During the Omicron Period

**DOI:** 10.3390/jcm15103702

**Published:** 2026-05-11

**Authors:** Atsuhito Suyama, Yuki Otsuka, Yasuhiro Nakano, Kazuki Tokumasu, Yasue Sakurada, Yui Matsuda, Hiroyuki Honda, Yoshiaki Soejima, Toru Hasegawa, Ryosuke Takase, Daisuke Omura, Kohei Oguni, Keigo Ueda, Fumio Otsuka

**Affiliations:** Department of General Medicine, Okayama University Graduate School of Medicine, Dentistry and Pharmaceutical Sciences, 2-5-1 Shikata-cho, Kita-ku, Okayama 700-8558, Japan; asuyama@s.okayama-u.ac.jp (A.S.); otsuka@s.okayama-u.ac.jp (Y.O.); y-nakano@okayama-u.ac.jp (Y.N.); tokumasu@okayama-u.ac.jp (K.T.); pzaf6h9w@s.okayama-u.ac.jp (Y.S.); phvw0350@okayama-u.ac.jp (Y.M.); ppgf1hrd@okayama-u.ac.jp (H.H.); yoshiakisoejima@s.okayama-u.ac.jp (Y.S.); pwcp0od9@s.okayama-u.ac.jp (T.H.); p4v05asb@okayama-u.ac.jp (R.T.); me20011@s.okayama-u.ac.jp (D.O.); oguni-gim@s.okayama-u.ac.jp (K.O.); p02n620b@okayama-u.ac.jp (K.U.)

**Keywords:** COVID-19, fatigue, IGF-I, long COVID, recovery

## Abstract

**Background:** Although several risk factors have been reported for long COVID (LC), reliable biomarkers for this illness remain lacking. Insulin-like growth factor (IGF)-I, a major mediator of growth hormones, plays an important role in metabolism, neuroprotection, and systemic homeostasis, and therefore may be useful in predicting the severity and prognosis of LC. **Methods:** This study included patients who visited a specialized clinic for long COVID between 2022 and 2025 during the Omicron period and had serum IGF-I measurements taken. IGF-I levels were expressed as age- and sex-adjusted standard deviation scores (IGF-I SD), and patients were classified into low (SD < −1.0), normal (−1.0 ≤ SD < 1.0), and high (SD ≥ 1.0) groups. Clinical characteristics, patient-reported outcomes, laboratory data, and follow-up duration were analyzed. **Results:** A total of 811 patients were included (median 42 years; 52.5% female). Compared with the normal group, the low-IGF-I group exhibited higher fatigue (FAS: 37.0 vs. 34.0; *p* < 0.05) and depressive (SDS: 50.0 vs. 49.0; *p* < 0.05) status. Multivariable linear regression analyses identified lower IGF-I SD as independently associated with higher scores of both FAS and SDS. IGF-I SD values showed negative correlations with ferritin (*ρ* = −0.125, *p* < 0.05) and TSH (*ρ* = −0.202, *p* < 0.01) and positive correlations with albumin (*ρ* = 0.227, *p* < 0.01) and FT4 (*ρ* = 0.165, *p* < 0.01). Among the 237 patients who completed follow-up, the median duration from the initial visit to recovery tended to be longer in the low-IGF-I group (221 days) compared with the normal (191 days) and high (109 days) groups, although these differences were not statistically significant overall. In patients aged < 50 years, the low-IGF-I group showed a relatively longer follow-up duration (*p* < 0.05). Furthermore, the low-IGF-I group had a longer time to recovery compared to the high-IGF-I group (*p* < 0.05), and this difference was more pronounced in patients under 50 years of age, with significant differences observed among the three IGF-I groups. **Conclusions:** Lower IGF-I levels in LC may be associated with greater fatigue and depressive symptoms and longer recovery time, particularly in younger patients. Further studies are needed to clarify the clinical significance of these findings.

## 1. Introduction

Long COVID (LC) is a clinical condition characterized by a wide range of persistent symptoms, including fatigue, headache, insomnia, and cognitive impairment (so-called “brain fog”), lasting for weeks to months after acute COVID-19 infection. These symptoms can significantly impair daily functioning and delay the return to normal social activities, posing a substantial clinical burden. Several factors associated with the development and persistence of LC have been reported, including female sex, older age, higher body mass index (BMI), severity of acute infection, comorbidities, and lack of vaccination [1]. However, these factors alone have limited predictive value, and reliable biomarkers capable of objectively predicting prognosis—particularly persistent fatigue and delayed recovery—have not yet been established. Since 2021, we have operated a COVID-19 aftercare (CAC) clinic as a regional referral center in western Japan, where we frequently encounter patients requiring months to years for recovery, highlighting the marked heterogeneity in clinical course.

Although the pathophysiology of LC is thought to be multifactorial—encompassing immune dysregulation, chronic inflammation, autoimmunity, autonomic dysfunction, endothelial dysfunction, and mitochondrial impairment—its overall mechanisms have yet to be fully elucidated [2,3,4]. In recent years, growing interest has focused on the role of the neuroendocrine system, particularly the hypothalamic–pituitary axis [5,6]. SARS-CoV-2 has been shown to impact both the hypothalamus and the pituitary gland, and such endocrine disturbances may persist into the chronic phase [7]. In our previous work, we identified delayed and prolonged hormonal responses in pituitary stimulation tests in patients with LC, especially in gonadotropin responses, which suggests hypothalamic dysfunction [8]. Furthermore, we found slightly reduced serum insulin-like growth factor (IGF)-I levels in these patients, implying possible involvement of the growth hormone (GH)/IGF-I axis.

A key pituitary hormone, GH, is also thought to play a role in this condition. Adult GH deficiency (AGHD) is characterized by symptoms such as fatigue, depression, reduced quality of life, and cognitive impairment—features that overlap substantially with those of LC. In AGHD, reduced GH secretion and decreased circulating IGF-I are associated with these clinical manifestations [9]. Based on these clinical and physiological similarities, it is plausible that dysregulation of the GH/IGF-I axis contributes to symptom persistence in LC. In addition, orthostatic intolerance—such as postural orthostatic tachycardia syndrome (POTS)—has been observed in younger individuals with LC [10]. We have further shown that GH secretion is markedly decreased in younger patients (<20 years) with LC who exhibit orthostatic dysregulation and POTS-like symptoms [11].

Despite these observations, the relationship between IGF-I levels and clinical manifestations—particularly fatigue and recovery trajectory—has not been sufficiently investigated in LC. IGF-I can be evaluated as an age- and sex-adjusted standard deviation (IGF-I SD) and can be easily measured in routine clinical practice, making it a potentially useful biomarker. Accordingly, this study sought to elucidate the relationship between serum IGF-I SD values and clinical manifestations as well as prognosis in patients with LC.

## 2. Patients and Methods

### 2.1. Study Population and Design

This single-center, retrospective observational study was carried out at the CAC outpatient clinic within the Department of General Medicine at Okayama University Hospital. We enrolled patients aged 10 years or older who attended the CAC clinic between January 2022 and September 2025, during the period when the Omicron variant was predominant, and who exhibited persistent symptoms for at least 4 weeks following COVID-19 infection, with available serum IGF-I measurements. LC was defined as the persistence of symptoms for a minimum of 4 weeks after infection that could not be attributed to other identifiable causes, as determined by the treating physician’s clinical judgment [12]. Inclusion in this study was determined based on clinical necessity.

### 2.2. Clinical Variables

Clinical information was extracted from electronic medical records, including age, sex, body mass index (BMI), smoking status, alcohol use, the severity of the acute COVID-19 infection [13], and vaccination history. Clinical symptoms and patient-reported outcomes were also collected, including the Fatigue Assessment Scale (FAS) [14,15], EuroQoL five dimensional questionnaire (EQ-5D) [16], Self-Rating Depression Scale (SDS) [17], and Frequency Scale for the Symptoms of Gastroesophageal Reflux Disease (FSSG) [18]. As a prognostic indicator, the duration from the initial visit to the CAC clinic to recovery was evaluated. Patients were considered “recovered” when their subjective and objective long COVID–related symptoms (e.g., fatigue, headache, dyspnea, cognitive impairment, and sleep disturbance) had improved or resolved to the extent that they no longer interfered with daily activities and no longer required follow-up, as determined by the attending physician. Symptom status was assessed through routine clinical interviews and physical examinations at each visit. This determination was based on real-world clinical practice rather than pre-defined standardized criteria. The time from the first to the final visit was defined as the follow-up duration.

### 2.3. Laboratory Data and Endocrine Evaluation

Blood samples were collected from patients as clinically indicated, primarily in the late morning after at least 5 min of rest in a seated position. Measured parameters included IGF-I, albumin (Alb), total protein (TP), triglycerides (TG), HDL cholesterol, LDL cholesterol, glucose-related markers, thyroid-stimulating hormone/thyrotropin (TSH), free thyroxine (FT4), adrenocorticotropic hormone/adrenocorticotropin (ACTH), cortisol, ferritin, and 50% hemolytic complement activity (CH50). Serum GH and IGF-I levels were measured using Elecsys GH and Elecsys IGF-I kits (F. Hoffmann-La Roche AG) with the Cobas 8000 auto-analyzer system (F. Hoffmann-La Roche AG, Basel, Switzerland) at the central laboratory of our hospital. Serum GH values below the detection limit (<0.03) were replaced with the detection limit value (0.03) for analysis. Similarly, CH50 values exceeding the upper detection limit (>60) were replaced with the upper detection limit value of 60. IGF-I values were expressed as age- and sex-adjusted standard deviation scores (IGF-I SD). Patients were classified into three groups as follows: low-SD group (IGF-I SD < −1.0), normal-SD group (−1.0 ≤ SD < 1.0), and high-SD group (SD ≥ 1.0) [19].

### 2.4. Statistical Analysis

Continuous variables are summarized as medians [interquartile range: IQR], while categorical variables are presented as counts and percentages. Comparisons between the two groups were conducted using the Mann–Whitney U test or Student’s *t*-test for continuous variables, as appropriate, and categorical variables were compared using the chi-square test or Fisher’s exact test. Differences among the three groups were assessed using the Kruskal–Wallis test for continuous variables and the chi-square test for categorical variables. Data normality was evaluated with the Shapiro–Wilk test. Associations between IGF-I SD values and laboratory parameters or follow-up duration were examined using Spearman’s rank correlation coefficient. Additional analyses were performed following stratification by sex. To assess differences in recovery status among IGF-I groups, the proportions of patients categorized as recovered versus not recovered were compared using the chi-square test. Among patients who achieved recovery, Kaplan–Meier analysis was performed to compare the duration from the initial visit to recovery among the IGF-I SD groups, and groups were compared using the log-rank test. Multivariable linear regression analyses were performed to evaluate the independent association between IGF-I SD and clinical outcomes (FAS and SDS), adjusting for age, sex, BMI, albumin, ferritin, thyroid function (TSH, FT4), and CH50. Because multiple comparisons were performed across symptoms, laboratory variables, and subgroup analyses, these analyses were considered exploratory in nature, and no formal correction for multiple testing was applied. All statistical analyses were performed using StataNow/SE 19.5 for Mac (StataCorp LLC, College Station, TX, USA), and a two-sided *p*-value < 0.05 was considered statistically significant. ChatGPT (GPT-5.3, OpenAI) was utilized to support language editing, paraphrasing, and proofreading of the manuscript.

### 2.5. Ethical Considerations

This study was carried out in accordance with the Declaration of Helsinki and received approval from the Ethics Committee of Okayama University Hospital (No. 2105-030). Given the retrospective nature of the study, the requirement for individual informed consent was waived. Instead, an opt-out procedure was implemented, in which study details were made publicly available and participants were allowed the opportunity to refuse participation.

## 3. Results

**Figure 1:**
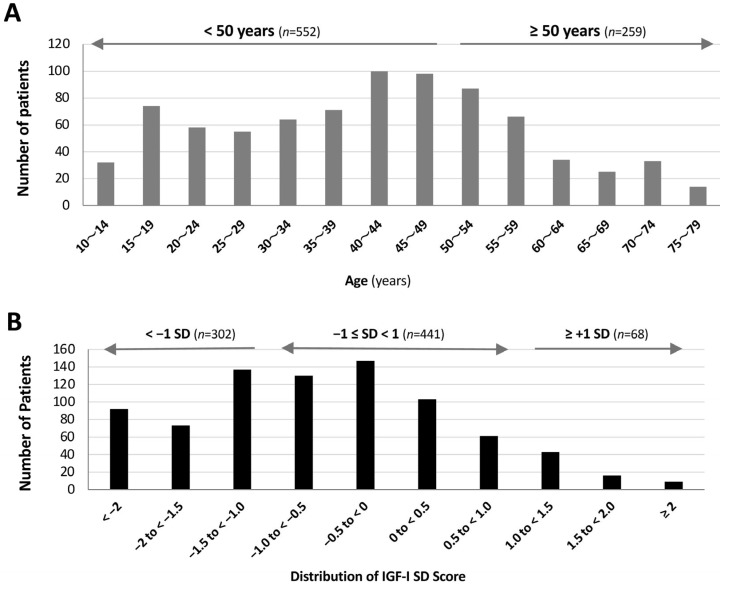
**Distribution of patient age and IGF-I SD scores in patients with long COVID.** (**A**) Age distribution of the study population. The number of patients in each age group is shown. Patients were stratified into two groups based on age: <50 years (*n* = 552) and ≥50 years (*n* = 259). (**B**) Distribution of IGF-I standard deviation (SD) scores. The number of patients in each IGF-I SD category is shown. Patients were classified into three groups based on IGF-I SD values: low (<−1.0, *n* = 302), normal (−1.0 to <1.0, *n* = 441), and high (≥1.0, *n* = 68). Ranges are expressed as lower bound-inclusive and upper bound-exclusive.

A total of 811 patients aged ≥10 years with LC who visited our hospital after January 2022 and had serum IGF-I measurements were included in the analysis. The median age was 42 years, and 52.5% of patients were female. The age distribution of the study population is shown in Figure 1A, with a peak in the 40–49-year age group. Patients were further stratified into two age groups for subsequent analyses: <50 years (*n* = 552) and ≥50 years (*n =* 259). Based on serum IGF-I SD values obtained at the initial CAC clinic visit, patients were classified into three groups: a low IGF-I SD group (*n* = 302), a normal IGF-I SD group (*n* = 441), and a high IGF-I SD group (*n* = 68; Figure 1B).

The baseline characteristics of the study population stratified by IGF-I SD levels are shown in Table 1. The median age in the low IGF-I group was 44 years, and 57.9% of patients were female; both differed significantly from those in the other groups (*p* < 0.01). The median BMI in the low IGF-I group was 22.2 kg/m^2^. A history of smoking was present in 30.7% of patients, and habitual alcohol consumption in 29.5%; these proportions did not differ among the three groups. COVID-19 vaccination (≥2 doses) was reported in 76.1% of patients in the low IGF-I group, comparable to the other groups. Regarding acute disease severity, 97.0% of patients had mild disease and 3.0% had moderate disease, with no severe cases observed in the low IGF-I group.

**Figure 2:**
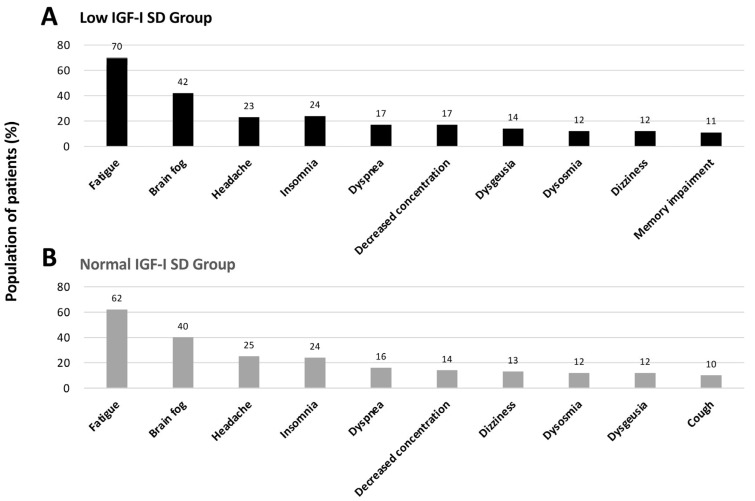
**Distribution of the most frequent symptoms in low- and normal-IGF-I SD groups.** (**A**) Low-IGF-I SD group and (**B**) normal-IGF-I SD group. Bar graphs show the top 10 most frequent symptoms in each group based on prevalence. The y-axis represents the percentage of patients, and symptoms are arranged in descending order of frequency within each group.

**Figure 3:**
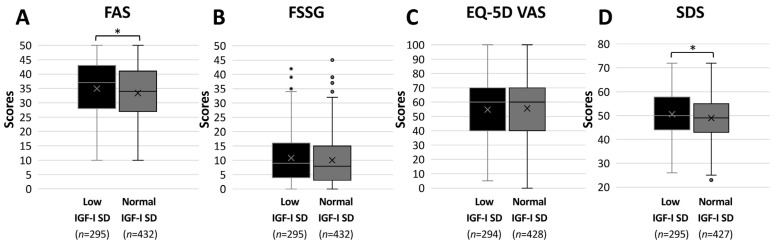
**Comparison of patient-reported outcome scores between low- and normal-IGF-I SD groups.** (**A**) Fatigue Assessment Scale (FAS) total score, (**B**) Frequency Scale for the Symptoms of Gastroesophageal Reflux Disease (FSSG) score, (**C**) EuroQol 5-Dimension Visual Analogue Scale (EQ-5D VAS), and (**D**) Self-Rating Depression Scale (SDS) score. Box plots show the distribution of each score in the low- and normal-IGF-I SD groups. The center line indicates the median, the box represents the interquartile range (IQR), and the whiskers represent values within 1.5 × IQR. The “×”symbol indicates the mean value. Comparisons between groups were performed using the Mann–Whitney U test. * *p* < 0.05 indicates statistical significance.

We then evaluated the characteristics of symptoms associated with LC according to IGF-I SD levels. In both the low-IGF-I and normal-IGF-I groups, fatigue was the most frequently reported symptom. Although the overall distribution of major symptoms was similar between groups, cognitive-related symptoms, such as memory impairment, appeared to be more frequent in the low-IGF-I SD group. (Figure 2). To further assess symptom burden quantitatively, patient-reported outcomes were compared between groups. The low IGF-I group showed significantly higher fatigue scores (FAS) compared with the normal IGF-I group (37.0 vs. 34.0, *p* = 0.028), and depression scores (SDS) were significantly higher in the low IGF-I group (50.0 vs. 49.0, *p* = 0.024). In contrast, no significant differences were observed in gastrointestinal symptom scores (FSSG) or quality of life (EQ-5D VAS; Figure 3).

We then examined the association between IGF-I levels and various laboratory parameters using Spearman’s rank correlation analysis. IGF-I SD scores showed significant negative correlations with ferritin (*ρ* = −0.125, *p* = 0.02) and TSH (*ρ* = −0.202, *p* = 0.001). In contrast, significant positive correlations were observed with albumin (*ρ* = 0.227, *p* < 0.001) and FT4 (*ρ* = 0.165, *p* = 0.002). Serum GH levels were also positively correlated with IGF-I SD values (*ρ* = 0.135, *p* < 0.001; Table 2).

Moreover, multivariable linear regression analyses identified IGF-I SD as independently associated with both FAS (*β* = −0.68, *p* = 0.033) and SDS (*β* = −0.82, *p* = 0.009) scores after adjustment for age, sex, BMI, albumin, ferritin, thyroid function, and CH50 (Table 3), with lower IGF-I SD being associated with higher symptom scores.

**Figure 4:**
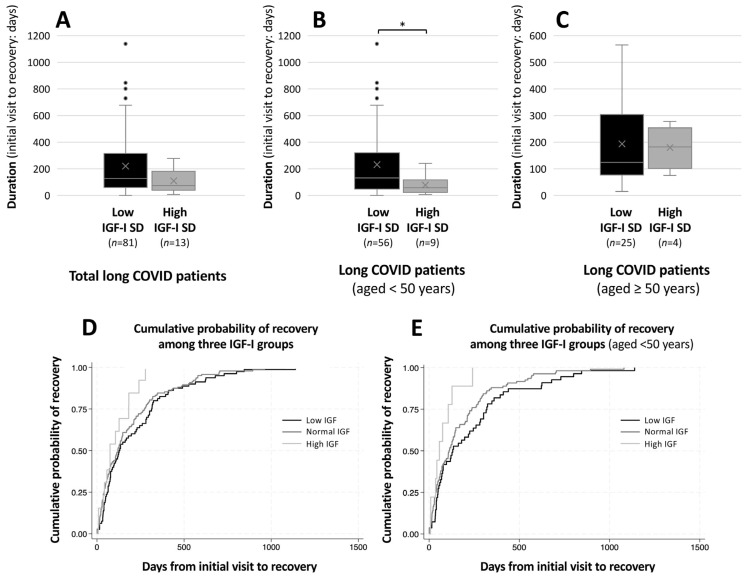
**Comparison of duration from initial visit to recovery according to IGF-I SD groups.** Duration was defined as the time from the initial visit to the CAC outpatient clinic to recovery. (**A**) Comparison of all patients who achieved recovery, (**B**) comparison of patients aged < 50 years, and (**C**) comparison of patients aged ≥ 50 years. Box plots show the distribution of duration (days) in the low- and high-IGF-I SD groups. The center line indicates the median, the box represents the interquartile range (IQR), and the whiskers represent values within 1.5 × IQR. The “×” symbol indicates the mean value. Comparisons between groups were performed using the Mann–Whitney U test. * *p* < 0.05 indicates statistical significance. Sample sizes are shown below each group. (**D**) Kaplan–Meier analysis among patients who achieved recovery in all age groups and (**E**) among patients aged < 50 years. Groups were compared using the log-rank test.

Finally, to evaluate the association between IGF-I levels and prognosis, we analyzed the duration of follow-up at the CAC clinic. Among the 237 patients who achieved recovery, the median follow-up duration was 221 days in the low-IGF-I SD group, 191 days in the normal-IGF-I SD group, and 109 days in the high-IGF-I SD group. Although these differences did not reach statistical significance, there was a trend toward a longer follow-up duration in the low IGF-I group compared with the other groups (low vs. normal IGF-I: *p* = 0.170, and low vs. high IGF-I: *p* = 0.090; Figure 4A). In an additional analysis stratified by age using a cutoff of 50 years, a significantly longer follow-up duration was observed in the low-IGF-I SD group among patients aged < 50 years (*p* = 0.041; Figure 4B), in which the median follow-up duration was 234 days in the low IGF-I group, 78 days in the high IGF-I group. In contrast, no significant differences were observed among patients aged ≥ 50 years (Figure 4C).

The proportion of patients who achieved recovery during the observation period (3 years and 9 months) differed among the groups, with rates of 26%, 33%, and 19% in the low, normal, and high IGF-I SD groups, respectively. The normal SD group had a significantly higher proportion of recovered patients compared with the other groups (chi-square test, *p* = 0.031). Among patients who achieved recovery, Kaplan–Meier analysis of the duration from the initial visit to recovery demonstrated that the low-IGF-I SD group had a longer time to recovery compared with the high-IGF-I SD group (log-rank test, *p* = 0.021; Figure 4D). Furthermore, this difference became more pronounced in patients aged < 50 years, in whom a significant difference was observed among the three groups (*p* = 0.018), with a particularly marked delay in recovery in the low-IGF-I SD group compared with the high-IGF-I SD group (*p* = 0.0086; Figure 4E).

## 4. Discussion

In the present study, patients with long COVID (LC) and low IGF-I levels showed greater fatigue, as assessed by the FAS, and more severe depressive symptoms, as assessed by the SDS. They also tended to require longer follow-up, suggesting a potential delay in recovery. These findings suggest that low IGF-I levels may be associated with a distinct clinical profile in LC.

IGF-I is a GH-dependent peptide, primarily produced in the liver, which exerts systemic endocrine effects as well as local actions in skeletal muscle and the central nervous system [20,21]. Its biological activity is tightly regulated through interactions with IGF-binding proteins (IGFBPs), and not only its circulating levels but also its bioavailability are considered important [22]. IGF-I plays a central role in maintaining physiological homeostasis, including protein anabolism, the preservation of muscle mass, mitochondrial function, neuroplasticity, and anti-inflammatory and neuroprotective effects [22,23]. Decreased IGF-I levels may lead to fatigue due to impaired muscle metabolism, the disruption of energy homeostasis, and neurocognitive dysfunction, including depression [20,22]. From this physiological perspective, the symptom profile observed in our study shares certain features with AGHD [24]. Importantly, multivariable linear regression analyses demonstrated that IGF-I SD was independently associated with both fatigue and depressive symptoms after adjustment for multiple clinical and laboratory variables, further supporting the robustness of this association.

With respect to infectious diseases, circulating IGF-I levels are generally reported to decrease following infection [25]. In acute COVID-19, lower serum IGF-I levels have been associated with disease severity and poorer outcomes, suggesting that IGF-I may reflect the severity of acute illness [26]. In contrast, longitudinal studies have reported no significant changes in IGF-I or IGF-binding protein acid-labile subunit (ALS) levels from the acute to the recovery phase of COVID-19 [27], indicating that the dynamics of IGF-I after infection remain unclear. In LC, endocrine abnormalities are generally mild, and one study reported that low IGF-I levels were present in only 3.6% of patients and were not significantly associated with clinical symptoms [5]. In contrast, we have previously demonstrated a negative correlation between serum ferritin and IGF-I levels in LC [28], as well as delayed gonadotropin responses suggesting hypothalamic dysfunction, accompanied by mildly reduced IGF-I levels [8]. Taken together, these findings suggest that the reduced IGF-I in LC may not simply represent a transient change following infection, but rather may reflect persistent pathophysiological alterations, potentially including hypothalamic dysfunction and metabolic dysregulation.

In this study, careful interpretation is required regarding the influence of age on IGF-I levels. IGF-I is known to decline with aging in a relatively linear manner up to approximately 50 years of age [19,20,29]. Therefore, age may modify the clinical interpretation of IGF-I levels, and similar IGF-I values may have different implications across age groups. In our cohort, the age distribution peaked at 40–49 years, with a median age of 42 years, consistent with previous epidemiological studies showing that LC predominantly affects middle-aged individuals [30]. Given this background, we conducted age-stratified analyses using 50 years as a threshold consistent with previous reports [19]. In patients under 50 years of age, the low IGF-I group showed a significantly longer follow-up period, whereas no such association was observed in patients aged 50 years or older. During the observation period (3 years and 9 months), recovery rates differed among IGF-I groups, with the normal IGF-I group showing a higher proportion of recovery. Kaplan–Meier analysis indicated that the low IGF-I group had a longer time to recovery compared with the high IGF-I group, particularly in patients under 50 years of age. However, these age-stratified findings should be considered exploratory and interpreted with caution. At the same time, interpretation of reduced IGF-I requires caution, as IGF-I reduction may act both as a cause of symptoms and as a consequence of systemic illness. On one hand, decreased IGF-I may directly contribute to fatigue and depressive symptoms through impaired neuroprotection and energy metabolism. On the other hand, chronic inflammation and poor nutritional status may also suppress GH secretion directly, leading to reduced IGF-I levels. In LC, these factors may interact and form a self-perpetuating cycle that contributes to symptom persistence.

Indeed, IGF-I is influenced by nutritional and metabolic status and is known to decrease under inflammatory and catabolic conditions [20]. In our study, IGF-I SD values showed negative correlations with ferritin and positive correlations with albumin, supporting this concept. On the contrary, serum GH levels were positively correlated with IGF-I SD values, supporting a physiological linkage with the GH/IGF-I axis. In acute illnesses such as sepsis and severe infections, inflammatory cytokines reduce hepatic GH receptor expression, leading to a state of “GH resistance” and decreased IGF-I production [25,31]. Similarly, in LC, persistent low-grade inflammation or prolonged post-acute effects may suppress the GH/IGF-I axis and contribute to metabolic stagnation. Furthermore, the dissociation between GH and IGF-I under conditions such as low BMI or hypoalbuminemia suggests that reduced IGF-I reflects not merely hormonal deficiency but also systemic metabolic maladaptation.

In addition, we observed that IGF-I SD values were negatively correlated with TSH and positively correlated with FT4, suggesting an association with thyroid function. Thyroid hormones are known to stimulate GH secretion and IGF-I production [20,32], and even mild hypothyroidism may suppress the GH/IGF-I axis [33]. The hypothalamus plays a central role in regulating multiple endocrine axes, including GH, thyroid, gonadal, and adrenal systems, and its dysfunction can manifest as multisystem endocrine dysregulation rather than isolated hormonal abnormalities [34]. In our previous studies, delayed gonadotropin responses suggested hypothalamic dysfunction in LC patients [8]. Taken together, these findings suggest that reduced IGF-I in LC may be associated with broader neuroendocrine dysregulation, potentially involving hypothalamic regulation. IGF-I may reflect underlying endocrine and metabolic status in LC and may warrant further investigation. While ferritin has been reported as a marker reflecting inflammation in LC [28], IGF-I may represent a more integrative indicator reflecting endocrine function, nutritional status, and metabolic reserve [20]. Notably, in patients younger than 50 years, reduced IGF-I was associated with prolonged recovery, suggesting that decreased endocrine resilience may contribute to delayed clinical improvement.

Nevertheless, this study has several limitations. First, this was a single-center retrospective study conducted in a specialized outpatient clinic, and causal relationships cannot be established. Although IGF-I measurements were routinely performed as part of the initial clinical assessment in the COVID-19 aftercare (CAC) clinic, selection bias cannot be completely excluded, particularly because patients with more persistent or severe symptoms may have been preferentially referred to this specialized setting. Second, the study lacked a non-long COVID control group, which limits the generalizability and specificity of the findings to long COVID. Third, the number of patients in the high-IGF-I SD group was relatively small, particularly in the age-stratified analyses, limiting the statistical reliability of these subgroup comparisons and the evaluation of potential non-linear associations. In addition, the age cutoff of 50 years was selected based on previously reported age-related changes in IGF-I levels; however, the appropriateness of this cutoff requires cautious interpretation, and the age-stratified analyses should therefore be considered exploratory. Longitudinal changes in IGF-I were not assessed in this study. Although the observation period extended up to 3 years and 8 months, recovery trajectories may continue to evolve over longer follow-up periods, and further long-term studies may therefore be warranted. Moreover, multiple comparisons were performed without formal correction for multiple testing; therefore, these exploratory findings should be interpreted with caution. In addition, the definition of recovery was based on clinical judgment in routine practice rather than standardized criteria or patient-reported outcomes, and unmeasured confounding may remain despite multivariable adjustment. Finally, the Kaplan–Meier analyses were limited to patients who achieved recovery and therefore may not fully reflect recovery trajectories in the entire cohort.

In conclusion, this study suggested that LC patients with low IGF-I levels may represent a subgroup characterized by increased fatigue, depressive symptoms, and delayed recovery. These findings support a potential involvement of the GH/IGF-I axis in the clinical course of LC and suggest that IGF-I warrants further investigation.

## Figures and Tables

**Table 1:** Baseline characteristics of long COVID patients grouped by serum IGF-I SD scores.

Variable	Low IGF-I SD (*n* = 302)	Normal IGF-I SD (*n* = 441)	High IGF-I SD (*n* = 68)	*p*-Value
Age, years, median [IQR]	44 [32–56]	41 [28–55]	41.5 [31–50.5]	0.006 **^(a)^
Female sex, n (%)	175 (57.9)	210 (47.6)	41 (60.3)	0.009 **^(b)^
BMI, kg/m^2^, median [IQR]	22.2 [19.7–25.7]	22.0 [19.5–25.0]	21.0 [18.5–24.0]	0.39 ^(a)^
IGF-I, ng/mL, median [IQR]	98 [72–124]	158 [121–196]	221.5 [169–273.5]	<0.001 **^(a)^
IGF-I SD, median [IQR]	−1.7 [−2.1–−1.4]	−0.2 [−0.6–0.2]	1.4 [1.1–1.6]	<0.001 **^(a)^
Smoking (past/current), n (%)	91 (30.7)	104 (23.9)	17 (25.4)	0.11 ^(b)^
Alcohol consumption, n (%)	87 (29.5)	105 (24.1)	14 (20.9)	0.16 ^(b)^
Vaccination (≥2 doses), n (%)	226 (76.1)	340 (78.0)	48 (70.6)	0.39 ^(b)^
Severity of COVID-19—Mild	288 (97.0)	428 (97.5)	66 (98.5)	
Severity of COVID-19—Moderate/Severe	9 (3.0)	11 (2.5)	1 (1.5)	0.76 ^(b)^

Continuous variables are presented as median [interquartile range (IQR)], and categorical variables as number (%). Differences among the three groups were assessed using the Kruskal–Wallis test for continuous variables and the chi-square test for categorical variables. ^(a)^ Kruskal–Wallis test; ^(b)^ chi-square test. Percentages were calculated excluding missing data. Missing data included 16 cases for BMI, 12 for smoking, 13 for alcohol consumption, 10 for vaccination status, and 8 for COVID-19 severity. ** *p* < 0.01 indicate statistical significance.

**Table 2:** Correlation between IGF-I SD scores and clinical laboratory parameters.

Variable	Spearman’s Rho (*ρ*)	*p*-Value
CH50	−0.011	0.83
Ferritin	−0.125	0.02 *
Alb	0.227	<0.001 **
TG	−0.069	0.20
LDL-C	−0.049	0.36
PPG	−0.100	0.06
Hemoglobin A1c	−0.008	0.89
TSH	−0.202	0.001 **
FT4	0.165	0.002 **
ACTH	0.027	0.61
Cortisol	−0.036	0.50
GH	0.135	<0.001 **

Associations between IGF-I SD scores and clinical laboratory parameters were evaluated using Spearman’s rank correlation coefficient. Spearman’s rho (*ρ*) and corresponding *p*-values are shown for each variable. * *p* < 0.05 and ** *p* < 0.01 indicate statistical significance. ACTH, adrenocorticotropic hormone; Alb, albumin; FT4, free thyroxine; GH, growth hormone; LDL-C, low-density lipoprotein cholesterol; PPG, postprandial plasma glucose; TG, triglycerides; TSH, thyroid-stimulating hormone.

**Table 3:** Multivariable linear regression analysis of factors associated with fatigue (FAS) and depressive (SDS) symptoms.

Variable	FAS (*β*-Coefficients [95% CI])	*p*-Value	SDS (*β*-Coefficients [95% CI])	*p*-Value
IGF-I SD	−0.68 [−1.31 to −0.06]	0.033 *	−0.82 [−1.44 to −0.21]	0.009 **
Age	−0.04 [−0.09 to 0.02]	0.182	0.02 [−0.03 to 0.07]	0.487
Male sex	−0.41 [−2.11 to 1.29]	0.638	−2.03 [−3.69 to −0.37]	0.017 *
BMI	0.07 [−0.09 to 0.24]	0.376	0.08 [−0.08 to 0.24]	0.318
Albumin	−0.09 [−2.43 to 2.24]	0.937	1.12 [−1.17 to 3.41]	0.338
Ferritin	−0.002 [−0.006 to 0.002]	0.367	−0.0004 [−0.005 to 0.004]	0.862
TSH	−0.004 [−0.25 to 0.24]	0.974	−0.06 [−0.30 to 0.18]	0.608
FT4	0.52 [−2.35 to 3.39]	0.723	1.02 [−1.79 to 3.82]	0.477
CH50	−0.004 [−0.10 to 0.09]	0.929	−0.02 [−0.12 to 0.07]	0.659

Multivariable linear regression analyses were performed to evaluate factors associated with fatigue and depressive symptoms, assessed by the Fatigue Assessment Scale (FAS) and Self-Rating Depression Scale (SDS), respectively. The model included IGF-I SD, age, sex, body mass index (BMI), albumin, ferritin, thyroid function (TSH and FT4), and CH50 as covariates. Results are presented as regression coefficients (*β*) with 95% confidence intervals (CI). *p*-values were calculated using two-sided tests. Negative *β*-coefficients indicate that higher values of the variable are associated with lower symptom scores. * *p* < 0.05 and ** *p* < 0.01 indicate statistical significance.

## Data Availability

The data presented in this study are available on request from the corresponding author (the data are not publicly available due to ethical restrictions).

## Abbreviations

ACTH, adrenocorticotropic hormone; BMI, body mass index; CAC, COVID-19 aftercare; EQ-5D, EuroQOL 5-dimension; FAS, fatigue assessment scale; FSSG, frequency scale for the symptoms of gastroesophageal reflux disease; FT4, free thyroxine; GH, growth hormone; IGF, insulin-like growth factor; IGFBPs, IGF-binding proteins; LC, long COVID; SDS, self-rating depression scale; TSH, thyroid-stimulating hormone.
